# Impact of HAART therapy on cognitive function in patients with HIV: a systematic review of randomized control trial

**DOI:** 10.1097/MS9.0000000000004821

**Published:** 2026-03-18

**Authors:** Mohammad Al Diab Al Azzawi, Arina Mohammed Alhamed, Hind Almajed, Wejdan Abdat Ahmed, Norah Nasser A. Alshahrani, Ibhar S. Idris, Omar Bader Aldayhani, Fadwa Fallatah, Rawan Hamdi Bedaiwi

**Affiliations:** a Faculty of Medicine, The National Ribat University, Khartoum, Sudan; b College of Medicine, Qassim University, Unayzah, Saudi Arabia; c College of Medicine, King Saud bin Abdulaziz University for Health Sciences, Riyadh, Saudi Arabia; d General Practitioner Department, King Salman bin Abdulaziz Hospital, Madinah, Saudi Arabia; e University of Bisha Medical College, Bisha, Aseer Region, Saudi Arabia; f College of Medicine, King Khalid University, Abha, Saudi Arabia; g College of Nursing, Ibn Sina National College for Medical Science, Makkah, Saudi Arabia; h Emergency Department, King Khalid Hospital, Tabuk, Saudi Arabia

**Keywords:** cognitive function, highly active antiretroviral therapy (HAART), HIV-associated neurocognitive disorders (HAND), randomized controlled trials (RCTs)

## Abstract

**Background:**

Cognitive impairment remains common in adults with HIV despite effective viral suppression on combination antiretroviral therapy. We systematically reviewed randomized trials evaluating the impact of different regimens or intensification strategies on cognitive outcomes.

**Methods:**

Following PRISMA, we searched PubMed, Scopus, Cochrane Library, and Web of Science from inception to 5 September 2025 for randomized controlled trials in adults that compared cognitive outcomes between combination antiretroviral regimens, placebo or no intensification. Eligible trials reported neuropsychological or cognitive test results. Risk of bias was assessed with the Cochrane ROB 2 tool. Owing to heterogeneity of cognitive measures, data were synthesized narratively.

**Results:**

Six trials (721 participants) met the criteria. Three evaluated initial regimens in antiretroviral-naïve participants and three tested maraviroc (MVC)- or dolutegravir-based intensification in virally suppressed individuals with neurocognitive impairment. Outcomes included global composite scores and domain-specific measures of memory, attention, executive function, processing/psychomotor speed, and motor function. Within-arm improvements were generally small; only one small pilot trial reported moderate cognitive benefit with MVC intensification. Larger trials did not show significant advantages of alternative or intensified regimens over standard therapy. Safety profiles were similar between arms.

**Conclusion:**

Available randomized evidence does not demonstrate a consistent additional cognitive benefit of alternative or intensified combination antiretroviral regimens beyond standard therapy with viral suppression. Given the small number and size of trials, modest regimen-specific effects in subgroups cannot be excluded. Future studies should use larger samples, harmonized outcome measures, and evaluate multimodal interventions combining optimized antiretroviral therapy with adjunctive pharmacological and non-pharmacological approaches.

## Introduction

**Figure 1.**
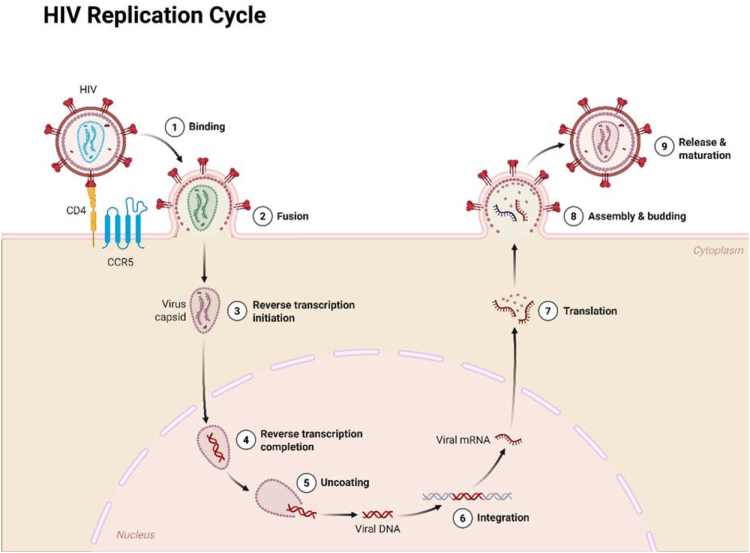
HIV replication cycle. Created by biorender.Com. Retrieved and modified from: https://www.Biorender.Com/template/hiv-replication-cycle.

**Figure 2.**
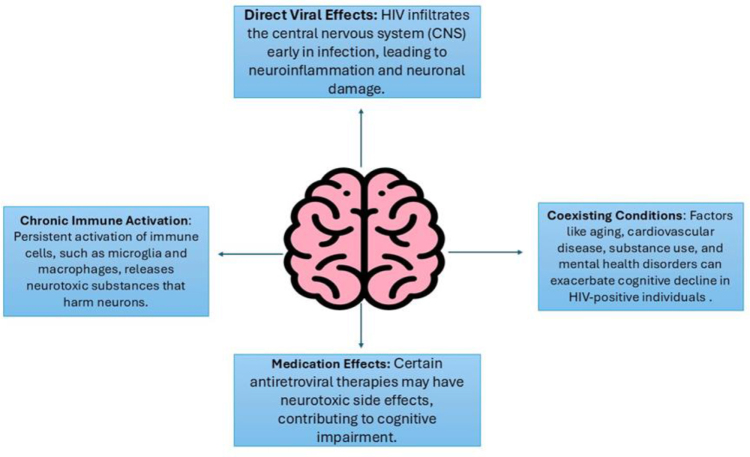
Most common causes contribute to cognitive impairment in HIV patients.

**Figure 3.**
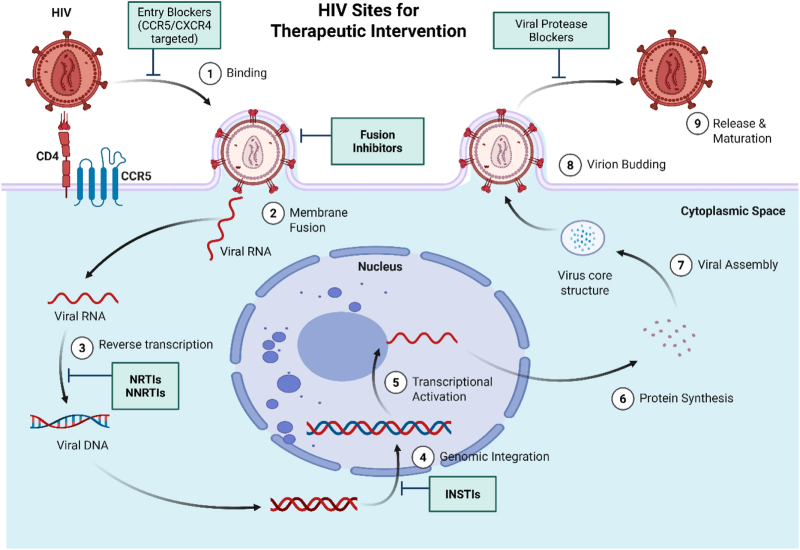
HIV sites for therapeutic intervention. Created by biorender.com. Retrieved and modified from: https://www.Biorender.Com/template/hiv-sites-for-therapeutic-intervention.

Human immunodeficiency virus (HIV) infection serves as a chronic viral systemic condition which specifically attacks CD4 + T cells and continues to worsen into the development of acquired immunodeficiency syndrome (AIDS)^[^1,2^]^. An illustration of HIV replication is illustrated in Figure 1. The HIV virus enters the central nervous system (CNS) to start neuroinflammation that produces neuronal damage resulting in HIV-associated neurocognitive disorders (HAND) with common causes that can cause neurocognitive disorders illustrated in Figure 2^[^3^]^. HAND identified by a spectrum of conditions ranging from asymptomatic neurocognitive impairment to mild neurocognitive disorder and HIV-associated dementia which require deficiency in at least two cognitive domains together with possible daily functioning deficits in severe cases^[^4,5^]^. Antiretroviral therapy (ART) serves as the foundation for HIV management by using drugs that block separate stages of HIV replication to maintain prolonged viral suppression and restore immune health consisting of three or more drugs from at least two drug classes, and it significantly decreased HIV-associated illnesses and death rates since the 1996 launch of highly active antiretroviral therapy (HAART)^[^6–8^]^. Over the past two decades, ART regimens have evolved to include integrase strand transfer inhibitors and fixed-dose combinations improving tolerability, simplifying dosing, and enhancing adherence, transforming HIV into a chronic and manageable condition^[^9,10^]^. HAART specifically refers to intensive combination regimens designed to maximize viral suppression and immune recovery by lowering both plasma and cerebrospinal fluid viral loads, HAART is theorized to ameliorate HIV-related neuroinflammation and neuronal injury, with potential benefits for cognitive function^[^9,11^]^. The initiation or intensification of HAART treatment has led to improved neurocognitive outcomes according to multiple randomized controlled trials (RCTs) which demonstrated positive changes in attention and memory together with executive function capabilities^[^12,13^]^. The research outcomes regarding HAART effects on cognition show conflicting results because some trials detected improvements, but different studies failed to find any measurable change in cognitive function^[^14–17^]^. An illustration for HIV sites for therapeutic intervention is shown in Figure 3. Despite evidence that HAART can improve neurocognitive performance, the magnitude and durability of these benefits across diverse patient populations remain unclear. Study heterogeneity stemming from differences in HAART regimens, neuropsychological assessment tools, and trial durations limits direct comparison of cognitive outcomes across RCTs. Moreover, the relative contributions of individual antiretroviral agents or drug classes to cognitive recovery are poorly characterized, impeding regimen-specific recommendations. Critical gaps also persist regarding HAART’s cognitive effects in aging populations, those with comorbidities (e.g., cardiovascular disease, substance use), and in resource-limited settings where regimen availability and monitoring differ significantly.

HIGHLIGHTS

- Systematic review of six randomized controlled trials (n = 721) on highly active antiretroviral therapy (HAART) and cognition in HIV adults
- Both treatment-naïve and virally suppressed cohorts were evaluated
- Only one small maraviroc pilot showed moderate cognitive benefit
- Larger trials found no cognitive advantage for intensified or alternative HAART
- Safety similar across regimens; no excess adverse events reported

We aimed to determine whether, among adults living with HIV virus receiving combination antiretroviral therapy, different regimens or intensification strategies improve global or domain-specific cognitive function compared with standard therapy or placebo. Our objectives were to (1) summarize randomized controlled trial evidence on changes in global neuropsychological performance z-score (NPZ); (2) describe effects on key cognitive domains (memory, attention, executive function, processing speed, and motor/psychomotor function); and (3) review associated functional, mood, and safety outcomes in relation to cognitive change.

## Method

### Protocol and registration

The Preferred Reporting Items for Systematic Reviews and Meta-Analysis (PRISMA) were followed in the conduct and reporting of this study^[^18^]^. This research protocol was registered on PROSPERO. This article is compliant with the TITAN Guidelines 2025^[^19^]^.

### Electronic search

Records gathered from PubMed, Scopus, Web of Science, and the Cochrane Library were imported into Rayyan reference library software to eliminate duplicate records^[^20^]^. The remaining articles were then screened based on their titles and abstracts, and studies meeting the inclusion criteria were subsequently assessed through full-text review. The screening process was independently carried out by reviewers; any discrepancies were resolved through discussion. The search strategy was (HIV OR AIDS OR “human immunodeficiency virus” OR “HIV-1 infection”) AND (HAART OR cART OR “highly active antiretroviral therapy” OR antiretroviral* OR “combination antiretroviral”) AND (cognit* OR “neurocognitive disorder” OR “neurocognitive impairment” OR “neuropsychological test*” OR “cognitive function”). The search covered all years from database inception to 5 September 2025 and was limited to studies published in English.

### Selection criteria

We defined eligibility using the PICOS framework. We included RCTs enrolling adults (≥18 years) with documented HIV infection. Eligible populations were either antiretroviral-naïve individuals initiating combination antiretroviral therapy or virally suppressed participants on stable combination therapy undergoing regimen modification or intensification. We did not specify a minimum duration of prior HAART exposure; for intensification trials, stability of antiretroviral therapy and viral suppression were defined according to each original trial protocol. Eligible interventions were HAART regimens or HAART intensification strategies compared with alternative antiretroviral regimens, placebo, or no intensification. Trials were required to report objective cognitive outcomes measured with standardized neuropsychological or cognitive test batteries, providing at least a global composite score and/or domain-specific measures such as attention, memory, executive function, processing, psychomotor speed, and motor function. We excluded non-randomized and observational studies, pediatric populations, trials without objective cognitive outcomes, single-arm or pharmacokinetic studies, and abstracts or conference proceedings without sufficient outcome data.

### Data extraction

The listed data were derived from original papers: (1) authors and publication year, (2) study design, (3) country, (4) sample characteristics (age, gender, BMI, CD4 count, mean (SD), (5) total number of participants, and (6) main result.

### Quality assessment

The quality of the RCTs included in this review was evaluated using the Risk of Bias 2 (ROB 2) tool^[^21^]^. This tool examines potential bias in five key domains: the randomization process, deviations from intended interventions, missing data, outcome measurement, and the selection of reported results. Each study was rated as having a low risk, some concerns, or a high risk of bias for each category. Independent reviewers did these assessments, resolving any disagreements through discussion. Visualization of the risk of bias was made using Robvis^[^22^]^.

### Data synthesis

We used a narrative synthesis to summarize cognitive outcomes by domain (memory, attention, executive function), detailing each trial’s HAART regimen, assessment tools, and follow-up. Because heterogeneity in measures and designs precluded quantitative pooling, we organized findings thematically, highlighting consistent trends and key discrepancies.

## Results

### Literature search

**Figure 4.**
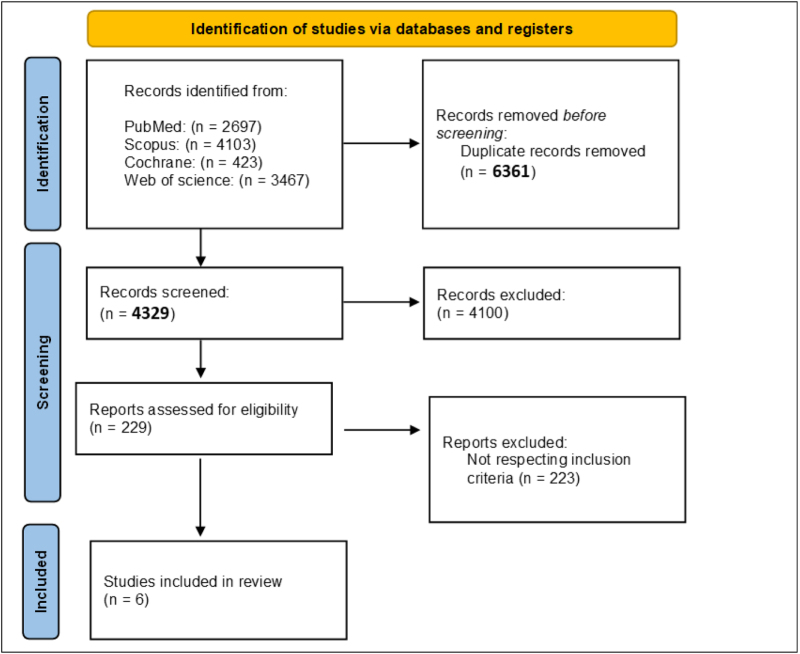
PRISMA flowchart summarizing identification and selection of studies.

A full search was performed across PubMed, Scopus, Cochrane Library, and Web of Science, to identify studies of HAART and cognition in HIV. After importing all records into reference management software, 6361 duplicates were removed. The remaining 4329 records were screened by title and abstract, excluding 4100 records that did not meet the inclusion criteria. Of the 229 full-text articles assessed, 223 were excluded for not meeting the criteria, leaving six studies for inclusion, as illustrated in Figure 4.

### The characteristic of the included studies

Six included trials published between 2010 and 2023 enrolled a total of 721 adults living with HIV across Europe, North America, Asia, Africa, South America, and Australia^[^12–17^]^. Three studies (Winston *et al* 2017; Robertson *et al* 2016; Winston *et al* 2010) focused on antiretroviral-naïve participants, comparing alternative HAART regimens (e.g., darunavir/ritonavir + raltegravir vs darunavir/ritonavir + TDF/FTC; maraviroc (MVC) + darunavir/ritonavir/FTC vs TDF + darunavir/ritonavir/FTC; and three different backbone regimens) over 48–96 weeks. Letendre *et al* (2023) evaluated 191 virally suppressed individuals with documented neurocognitive impairment randomized to dolutegravir (DTG) + MVC or DTG + placebo for up to 96 weeks. Gates *et al* (2016) piloted addition of MVC to stable cART in 14 men with HIV-associated neurocognitive disorder over 48 weeks. Shikuma *et al* (2023) conducted a double-blind 48-week trial in virally suppressed adults with mild HAND randomized to maraviroc intensification or placebo. MVC intensification did not improve global or domain NPZ versus placebo and viral suppression and safety were maintained in both groups.

All trials measured standardized neuropsychological outcomes such as NPZ composite scores, global deficit scores, and domain-specific z-scores after 48–96 weeks. Across studies, most regimens achieved viral suppression, but only the small Australian pilot demonstrated medium-to-large effect sizes favoring MVC for global cognitive improvement; the larger RCTs found no statistically significant differences between intervention and control arms, as illustrated in Tables 1 and 2.

**Table 1:** Summary of the included studies.

Study ID	Design	N	Core population	Intervention → control	Primary outcome	Follow-up	Key finding
Gates *et al* (2016)	Pilot RCT, double-observer-blinded	17	Virally suppressed men with HAND	Maraviroc add-on → No MVC add-on	Global neurocognitive z-score	12 months	Medium–large effect sizes favoring MVC at 6 months (d = 0.77) and moderate at 12 months (d = 0.55), but confidence intervals included zero and results were not statistically definitive.
Letendre *et al* (2023)	Phase 4 RCT, double-blind	191	Stable ART ≥ 6 mo, HAND	DTG + MVC/DTG + placebo → dual placebo	Total neurocognitive z-score	96 wk	No MVC benefit at 48 or 96 wk
Robertson *et al* (2016)	Phase 2 RCT, double-blind	262	ART-naïve, CCR5-tropic HIV	MVC + DRV/r/FTC → TDF + DRV/r/FTC	Global deficit & total z-scores	48 wk	Equal cognitive gain; 49 % normalized in both arms
Shikuma *et al* (2023)	Double-blind RCT	49	Suppressed ART ≥ 1 yr, mild HAND	MVC intensification → Placebo intensification	Global & domain NPZ	48 wk	No overall benefit; small, non-sig. memory gain
Winston *et al* (2010)	Open-label RCT	30	ART-naïve, no NCI	(1) TDF/FTC + EFV; (2) TDF/FTC + ATV/r; (3) TDF/FTC + ZDV/ABC (active-arm comparison)	Neurocognitive tests & brain MRS	48 wk	Arm 3 superior on executive function; all arms suppressed virus
Winston *et al* (2017)	Open-label RCT, non-inferiority	208	ART-naïve, CD4⁺ < 500 or symptomatic	DRV/r + RAL → DRV/r + TDF/FTC	DSST & global NPZ	96 wk	No difference (ΔNPZ 0.28 vs 0.21; *P* = 0.37)

**Table 2:** Baseline characteristics of the included studies.

Author (year)	Arm	N	Age, years	Male n (%)	CD4 cells/µL	Education, years	White n (%)	Black n (%)
Gates *et al* (2016)	Maraviroc	9	52.2 (3.7)	9 (100)	499 (489.5)	12.3 (2.8)	N/A	N/A
	Control	5	60.0 (9.4)	5 (100)	980 (493)	11.6 (2.3)	N/A	N/A
Letendre *et al* (2023)	DTG + MVC	61	52 (8)	43 (70.5)	726 (331)	N/A	N/A	N/A37 (61)
	DTG + placebo	67	52 (9)	44 (66)	703 (278)	N/A	N/A	N/A30 (45)
	Dual placebo	63	52 (7)	48 (76)	681 (294)	N/A	N/A	N/A30 (48)
Robertson *et al* (2016)	MVC	119	34.33 (12.01)	N/A	396.7 (160.4)	N/A	N/A	N/A
	TDF	111	33.67 (12.02)	N/A	396.7 (160.4)	N/A	N/A	N/A
Shikuma *et al* (2023)	MVC intensification	31	55.7 (7.4)	15 (48)	707.7 (374.1)	13.0 (1.5)	9 (29)	1 (3)
	Placebo intensification	17	55.7 (4.4)	9 (52)	707.7 (341.8)	14.0 (1.5)	6 (35)	0 (0)
Winston *et al* (2010)	TDF/FTC + EFV	9	35 (11)	N/A	235 (56)	N/A	4 (44)	N/A
	TDF/FTC + ATV/r	9	37 (10)	N/A	194 (84)	N/A	2 (22)	N/A
	TDF/FTC + ZDV/ABC	12	33 (19)	N/A	222 (109)	N/A	5 (42)	N/A
Winston *et al* (2017)	DRV/r + TDF/FTC	115	40 (12.8)	102 (89)	344.7 (86.3)	12.7 (3.8)	101 (88)	10 (9)
	DRV/r + RAL	93	37.7 (11.3)	87 (94)	340.3 (85.1)	13.3 (5.3)	77 (83)	11 (12)

### Quality assessment of the included studies

**Figure 5.**
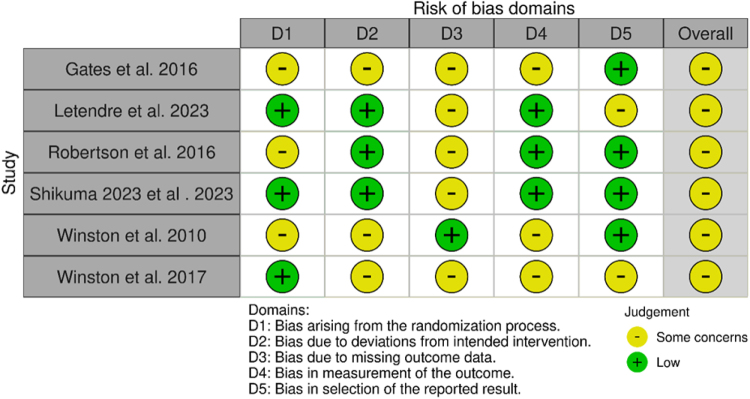
The bias-risk assessment diagram of the included studies.

**Figure 6.**
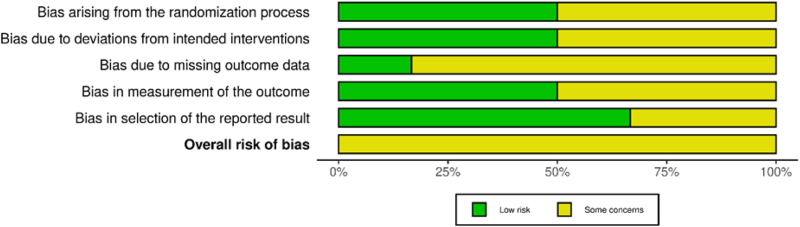
The bias evaluation bar graph of the included studies.

The risk of bias was assessed using the Cochrane ROB 2 tool across five domains. All included studies (Winston *et al* 2017; Letendre *et al* 2023; Robertson *et al* 2016; Winston *et al* 2010; Gates *et al* 2016, and Shikuma *et al* 2023) were judged to have an overall risk of some concerns^[^12–17^]^. Winston *et al* (2017) had a low risk in the randomization process but showed some concerns in deviations from intended interventions, missing outcome data, measurement of the outcome, and selection of the reported result. Letendre *et al* (2023) demonstrated low risk in randomization, deviations, and outcome measurement but had some concerns regarding missing data and selective reporting. Robertson *et al* (2016) had some concerns in randomization and missing data, while other domains were at low risk. Winston *et al* (2010) showed some concerns across randomization, deviations, and outcome measurement, but low risk in missing data and reporting. Similarly, Gates *et al* (2016) showed some concerns in all domains except for the selection of reported results, where the risk was low. Shikuma *et al* (2023) showed low risk in randomization, deviations from intended interventions, outcome measurement, and selective reporting, but some concerns regarding missing outcome data. Overall, concerns were most frequently related to deviations from intended interventions, missing outcome data, and selective reporting, as illustrated in Figures 5 and 6.

## Narrative synthesis

### Change in global NPZ

Six RCTs reported change in NPZ after antiretroviral intensification or modification.

Three trials evaluated intensification in virally suppressed adults with neurocognitive impairment.

Shikuma *et al* (2023) randomized 49 participants in a 2:1 ratio to MVC intensification (n = 32) or placebo (n = 17) on a background of stable ART for 48 weeks. Mean change in global NPZ from baseline to week 48 was 0.025 ± 0.475 in the MVC arm and 0.097 ± 0.250 in the placebo arm; the between-group difference in change was not statistically significant.

Gates *et al* (2016) randomized 17 patients with HAND to MVC intensification (n = 9) or continuation of existing ART (control, n = 8). Fourteen participants (9 MVC, 5 control) completed the 12-month visit. For the global CogState z-score, the arm × time interaction was statistically significant at 6 months with a regression coefficient b = − 0.10 (standard error 0.04, 90% CI −0.18 to −0.03; *P* < 0.03), corresponding to an effect size d = 0.77 (90% CI −0.19 to 1.71). At 12 months, the coefficient was b = − 0.01 (standard error 0.05, 90% CI −0.09 to 0.06; *P* = 0.77), corresponding to d = 0.55 (90% CI −0.47 to 1.55).

Letendre *et al* (2023) conducted the A5324 trial, in which 191 virally suppressed participants with neurocognitive impairment were randomized to DTG + MVC, DTG + placebo, or dual placebo for 96 weeks. The primary endpoint was change in normalized total neurocognitive z-score at week 48. Total z-scores increased over time in all three arms. Adjusted analyses at week 48 and later visits showed no statistically significant differences in change in total z-score between DTG + MVC, DTG + placebo, and dual placebo.

Three trials evaluated initial or alternative ART regimens.

Robertson *et al* (2016) conducted ACTG A5303, in which 262 ART-naïve participants were randomized to MVC-containing versus TDF-containing regimens. At week 48, the median change in total neuropsychological z-score was 0.3 (interquartile range [IQR] 0.0–0.5) in the MVC arm and 0.3 (IQR 0.1–0.5) in the TDF arm; there were no statistically significant between-arm differences in change in total z-score or global deficit score.

Winston *et al* (2017) reported the NEAT 001/ANRS 143 cognitive substudy, in which participants initiating darunavir/ritonavir-based therapy were randomized to an NRTI-sparing regimen (darunavir/ritonavir + raltegravir) or a standard regimen (darunavir/ritonavir + tenofovir/emtricitabine). At week 96, composite NPZ scores had increased from baseline in both arms. The adjusted between-arm difference in NPZ change at week 96 was small and not statistically significant (*P* > 0.05).

Winston *et al* (2010) reported the Altair cognitive substudy, in which 30 ART-naïve participants were randomized across three first-line regimens. All three regimens were associated with improved performance; some measures, particularly reaction time and executive function, improved significantly more with the ZDV + ABC regimen (arm 3) vs EFV-based therapy.

Across these six trials, global composite neuropsychological scores improved over follow-up within treatment arms, and the reported between-arm differences in change (where given numerically) were small and not statistically significant.

### Domain-specific cognitive performance

Five trials reported domain-specific cognitive outcomes. Shikuma *et al* (2023) calculated domain NPZ scores for executive function, processing speed, attention, learning and memory, and fine motor performance. Over 48 weeks, mean domain z-scores changed modestly in both MVC and placebo arms. Initial unadjusted analyses indicated some differences (for example, in learning and memory), but after covariate adjustment and correction for multiple comparisons, there were no statistically significant between-arm differences in change in any cognitive domain. In the trial by Gates *et al* (2016), the CogState battery assessed psychomotor function, attention, learning, and working memory. Domain-level effect sizes for change at 6 and 12 months generally favored MVC, in line with the global z-score findings, but the 90% CI for individual domains included zero, consistent with the small sample size. Robertson *et al* (2016) reported that speed of processing, executive function, verbal learning, verbal memory, and motor function improved from baseline to week 48 in both MVC- and TDF-containing arms. The magnitude of mean change in domain scores was similar between arms, and formal comparisons did not identify statistically significant between-group differences in domain-level change. Winston *et al* (2017) found that scores on individual domain tests (for example, Trail Making Test A and B for attention and executive function) improved between baseline and week 96 in both the NRTI-sparing and standard arms. A statistically significant arm difference was reported for attention (Trail Making Test A) with greater improvement in the raltegravir arm (*P* = 0.0499), but other domains did not show statistically significant between-arm differences in change.

Winston *et al* (2010) reported that performance across domains such as reaction time, executive function, and learning improved during 48 weeks in all three regimens. In some tests, one arm showed larger mean change than another, but these effects were not consistent across the full domain battery and were not accompanied by large differences in global performance.

Overall, domain-specific data demonstrate that attention, memory, executive function, processing or psychomotor speed, and motor function tended to improve over time within arms across studies, with between-arm differences generally small and, when formally tested, usually non-significant; apart from isolated findings in the small MVC pilot trial and a single attention measure in the raltegravir arm of NEAT 001/ANRS 143, no regimen showed a reproducible advantage in any specific cognitive subdomain.

### Biomarkers and ancillary pathophysiological measures

Three trials reported biomarkers or neuroimaging measures alongside neuropsychological outcomes.

Gates *et al* (2016) measured single-voxel ^1^H-MRS metabolite concentrations and CSF inflammatory markers at baseline, 6 months, and 12 months. Despite effect sizes favoring MVC for global neurocognitive z-scores (d = 0.77 at 6 months and 0.55 at 12 months with 90% CIs that included zero), statistical analyses did not detect significant treatment-related changes in ^1^H-MRS metabolites or CSF biomarkers when MVC and control arms were compared. Letendre *et al* (2023) reported that some immunologic biomarkers (e.g. CD4/CD8 counts, MIP-1β) differed between arms, but these changes were not associated with neurocognitive or functional outcomes.

Winston *et al* (2010) measured N-acetylaspartate/creatine (NAA/Cr) ratios by ^1^H-MRS in multiple brain regions. NAA/Cr increased from baseline to week 48 in all three treatment arms. In some regions, percentage increases in NAA/Cr differed between arms and reached statistical significance, but these regional differences were not consistently reflected in global neuropsychological outcomes.

### Functional status and daily living

Functional status, primarily using instrumental activities of daily living (IADL) scales, was reported in two intensification trials.

Gates *et al* (2016) measured functional decline using a standard Independence of Activities of Daily Living (IADL) questionnaire. IADL scores were collected at baseline and follow-up. Over 6 and 12 months, scores showed small changes or remained stable in both the MVC intensification and control arms, and no statistically significant between-arm differences in change were reported. Letendre *et al* (2023) assessed functional status using the revised Lawton and Brody IADL scale. IADL scores were measured repeatedly up to week 96. Mean IADL values increased modestly over time in all three arms (DTG + MVC, DTG + placebo, dual placebo). Mixed repeated-measures models comparing change over time between arms did not show statistically significant differences in IADL trajectories. Across these two trials, functional status as measured by IADL-type scales showed small within-arm improvements or stability over time, and no study demonstrated a statistically significant functional advantage of intensification compared with control.

### Depression and psychological symptoms

Depressive symptoms and psychological distress were systematically assessed in three intensification studies. Letendre *et al*. (2023) measured depressive symptoms by BDI-II repeatedly to week 96. Mean depression scores decreased over time across all arms. Adjusted analyses of change showed no statistically significant differences between DTG + MVC, DTG + placebo, and dual-placebo groups at week 48 or at subsequent visits. Shikuma *et al* (2023) administered the Beck Depression Inventory-II (BDI-II) at baseline and week 48. Baseline BDI-II scores were in the mild range. Mean BDI-II scores decreased slightly in both MVC and placebo arms over 48 weeks, and between-arm differences in change were not statistically significant. Gates *et al* (2016) used the Depression Anxiety Stress Scales (DASS-21). Mean depression, anxiety, and stress subscale scores showed small changes at 6 and 12 months in both MVC and control arms, and no statistically significant differences in change between arms were reported.

Overall, depression and psychological symptom measures showed modest within-arm improvement or stability, without statistically significant effects of intensification compared with control regimens.

### Safety and tolerability

Safety and tolerability were reported in all six trials. In Letendre *et al* (2023), 15 of 191 participants (8%) discontinued study drug due to adverse events over 96 weeks. The proportion of participants discontinuing for adverse events did not differ significantly between DTG + MVC, DTG + placebo, and dual placebo arms (*P* = 0.17). The overall incidence of adverse events, serious adverse events, and laboratory abnormalities was similar across arms.

Shikuma *et al* (2023) reported that 49 participants received MVC or placebo for 48 weeks. The frequency and type of adverse events, including serious adverse events, were comparable between MVC and placebo arms, and there were no statistically significant differences in discontinuations due to toxicity.

Gates *et al* (2016) found that MVC intensification was well tolerated. No treatment-related serious adverse events were reported, and non-serious adverse event profiles were similar in MVC and control arms.

Robertson *et al*. (2016) reported that 262 ART-naïve participants received MVC- or TDF-containing regimens. Rates of adverse events, serious adverse events, and treatment discontinuations were similar between arms, with no statistically significant differences in safety outcomes.

Winston *et al* (2010, 2017) reported that first-line regimens including NRTI-sparing and conventional NRTI-containing combinations showed broadly comparable safety profiles. Serious adverse events and discontinuation rates did not differ substantially between randomized arms, and no consistent statistically significant between-arm differences in major safety endpoints were observed.

Across all trials, intensification strategies and alternative ART regimens were generally well tolerated, and safety outcomes did not differ significantly between experimental and comparator arms.

## Discussion

The current systematic review evaluated the effects of HAART on cognitive performance in adults with HIV, and it synthesized six RCTs published between 2010 and 2023. The population of these studies was diverse geographically and compared various HAART regimens and assessed their cognitive potential using standardized measures of neuropsychological testing.

## Cognitive outcomes and HAART regimens

In general, the available evidence in the studies included indicates mixed cognitive effects of different HAART strategies. Three studies of antiretroviral-naive individuals compared early HAART regimes and found no substantial benefit of one combination over another. Winston *et al* have shown similar NPZ between an NRTI sparing regimen (darunavir/ritonavir plus raltegravir) and conventional regimen (darunavir/ritonavir plus tenofovir/emtricitabine) at 96 weeks^[^14^]^. On the same note, Robertson *et al* reported no significant cognitive differences between MVC-based and tenofovir-based regimens, with similar global neuropsychological improvement^[^13^]^. Nevertheless, Winston *et al* did note certain cognitive domain benefits with an abacavir/zidovudine-based regimen compared to efavirenz-containing regimens, indicating small but potentially significant cognitive differences about regimen composition^[^16^]^.

## Intensification strategies

Intensification studies with MVC or DTG aimed to enhance cognitive outcomes in patients already virally suppressed on stable HAART, particularly those with established neurocognitive impairment. However, these trials yielded generally negative results. Shikuma *et al* showed negligible overall cognitive improvement following MVC intensification compared to placebo over 48 weeks^[^15^]^, a finding by Letendre *et al*, who reported no significant neuropsychological or functional benefits from DTG-based intensification^[^17^]^. Conversely, the small pilot study by Gates *et al* indicated moderate cognitive improvement at 6 and 12 months post-MVC intensification, though these results should be cautiously interpreted given the limited sample size and study design^[^12,17^]^.

## Functional and psychological outcomes

Notably, functional status measured by IADL scales in Gates *et al* and Letendre *et al* was consistent in showing that despite within-arm improvements in cognitive test scores, there were no significant changes in real-world functioning attributable to intensification strategies^[^12,17^]^. Moreover, psychological tests failed to show any substantial improvement of depressive symptoms and psychological distress that could be ascribed to an increase in HAART, indicating that there was a minor psychological effect of the interventions^[^13,17^]^.

## Safety and tolerability

A consistent finding across included studies was the favorable safety profile of intensified HAART regimens. MVC- and DTG-based regimens were generally well-tolerated, exhibiting no substantial differences in serious adverse events or discontinuation rates compared to placebo or standard-of-care regimens. This reinforces the acceptable safety profile of HAART intensification, despite the observed lack of substantial cognitive benefit^[^12,15,17^]^.

Our results are consistent in general with previous systematic reviews and meta-analyses examining neuropsychological performance in patients with HIV under treatment with HAART. In line with the existing evidence, the large-scale cognitive gain seems to be minimal compared to different primary or intensified regimens^[^23–25^]^. A systematic review by Cysique and Brew also came to the same conclusion that HAART tends to stabilize cognition, but does not significantly reverse impairment, especially in patients who are already virally suppressed^[^23^]^. Similarly, Nightingale *et al* found little evidence that any HAART regimen or intensification strategy is better in neurocognitive outcomes and this further supports the idea that regimen choice does not directly affect neurocognitive outcomes^[^25^]^. In contrast, the favorable cognitive benefits seen in the small pilot trial conducted by Gates *et al* have not been reproduced in larger randomized trials highlighting the necessity of careful interpretation because of sample size and design differences^[^12,23^]^.

Beyond these earlier reviews, our study adds an updated synthesis restricted to RCTs of specific HAART regimens and intensification strategies, incorporates more recent trials, and provides a structured analysis across global and domain-specific cognitive outcomes, functional status, mood, biomarkers, and safety.

## Mechanisms of persistent cognitive impairment

Persistent cognitive impairment in virally suppressed individuals is likely to be multifactorial. Chronic low-grade neuroinflammation and residual CNS viral reservoirs may continue despite effective systemic suppression, and the lack of consistent between-group changes in inflammatory or neuronal injury biomarkers across trials suggests that regimen intensification has limited impact on these processes. In many participants, long-standing infection and pre-existing HIV-associated neurocognitive disorder mean that irreversible structural damage to white matter and cortical networks may restrict the potential for cognitive recovery. Potential neurotoxic effects and cumulative exposure to certain antiretroviral agents, together with aging, vascular risk factors, and other comorbidities, may further contribute to ongoing cognitive dysfunction. These mechanisms help to explain why modifications of antiretroviral regimens alone produced only modest cognitive changes in the included randomized trials.

## Clinical implications and future directions

Clinically these findings suggest limited utility of regimen intensification specifically for cognitive improvement in HIV patients with suppressed viral loads. Despite promising pilot outcomes larger controlled trials failed to replicate meaningful cognitive improvements. Therefore, clinical management decisions should prioritize regimens primarily for sustained viral suppression and tolerability rather than specific cognitive benefits. Future studies should explore targeted interventions including adjunctive therapies or non-pharmacological approaches, to address persistent cognitive impairments in HIV-positive individuals with standard ART.

## Strengths, limitations, and future directions of this review

The main strength of this systematic review is that it includes RCTs conducted in various international contexts, which increases the applicability of the results. Nonetheless, several limitations should be noted. There was substantial variability in neuropsychological outcome measures, study populations, and follow-up durations, and sample size was small in many trials. Only six RCTs, including 721 participants, met the inclusion criteria, which limits the statistical power and generalizability of our findings. Using the RoB 2 tool, all trials were judged as having at least “some concerns” risk of bias, and residual bias related to outcome measurement, attrition, or selective reporting cannot be excluded. Because only six trials were available, we did not construct funnel plots or apply formal statistical tests for publication bias, which are typically recommended only when a larger number of studies are available, and selective non-publication of small or null trials remains possible.

Future randomized studies should therefore enroll larger and more diverse samples, use harmonized cognitive test batteries with agreed domain definitions, include functional and patient-reported outcomes, extend follow-up beyond 1–2 years, and incorporate biomarkers and neuroimaging to clarify mechanisms and identify subgroups most likely to benefit from multimodal interventions.

## Conclusion

The available randomized evidence suggests that initiation of combination antiretroviral therapy is associated with modest improvements in cognitive performance, but switching between contemporary first-line regimens or intensifying suppressive therapy with MVC or DTG has not been shown to provide a clear additional cognitive benefit. These findings should be interpreted cautiously given the small number and size of trials and the heterogeneity of cognitive outcomes, and modest regimen-specific effects in particular subgroups cannot be excluded. Clinically, antiretroviral regimen changes should therefore not be made solely with the expectation of improving cognition; instead, management of HIV-associated cognitive impairment should prioritize early and sustained viral suppression, optimization of comorbidities, and evaluation of multimodal and adjunctive interventions in adequately powered studies.

## Data Availability

Not applicable.

## Research registration unique identifying number (UIN)

CRD42024545843.

## Provenance and peer statement

Not commissioned, externally peer-reviewed
